# Genetic analysis of DNA methylation in dyslipidemia: a case-control study

**DOI:** 10.7717/peerj.14590

**Published:** 2022-12-19

**Authors:** Shuai Liu, Yang Li, Xian Wei, Dilare Adi, Yong-Tao Wang, Min Han, Fen Liu, Bang-Dang Chen, Xiao-Mei Li, Yi-Ning Yang, Zhen-Yan Fu, Yi-Tong Ma

**Affiliations:** 1 First Affiliated Hospital of Xinjiang Medical University, Urumqi, China; 2 Xinjiang Key Laboratory of Cardiovascular Disease Research, Urumqi, China

**Keywords:** Coronary artery disease, Dyslipidemia, DNA methylation, *GRINA*, Haplotype, CpG

## Abstract

**Background:**

Coronary heart disease has become the leading cause of death in developed countries, and dyslipidemia is closely associated with the risk of cardiovascular disease. Dyslipidemia is caused by the abnormal regulation of several genes and signaling pathways, and dyslipidemia is influenced mainly by genetic variation. *AMFR, FBXW7, INSIG1, INSIG2*, and *MBTPS1* genes are associated with lipid metabolism. In a recent GWAS study, the *GRINA* gene has been reported to be associated with dyslipidemia, but its molecular mechanism has not been thoroughly investigated. The correlation between the DNA methylation of these genes and lipid metabolism has not been studied. This study aimed to examine the relationship between the DNA methylation of these genes and the risk of dyslipidemia by comparing the methylation levels of dyslipidemia and control samples.

**Methods:**

A case-control research method was used in this study. The patient’s blood samples were collected at the Heart Center of the First Affiliated Hospital of Xinjiang Medical University. In the Xinjiang Han population, 100 cases of hyperlipidemia and 80 cases of the control group were selected. The two groups were age and gender-matched. Quantitative methylation analysis of CpG sites in the gene promoter regions of six genes was performed by Solexa high-throughput sequencing.

**Results:**

The DNA methylation levels of 23 CpG sites in six genes were shown to be associated with hyperlipidemia, and a total of 20 DNA methylation haplotypes showed statistically significant differences between the two groups. When compared with the control group, the dyslipidemia group had significantly higher levels of methylation in the *GRINA* gene (2.68 *vs* 2.36, *P* = 0.04). Additionally, we also discovered a significant methylation haplotype of GRINA (*P* = 0.017).

**Conclusion:**

The findings of this study reveal that the DNA methylation of *GRINA* increases the risk for dyslipidemia in humans.

## Introduction

Coronary artery disease (CAD) is a major contributor to global mortality (GBD 2013 Mortality and Causes of Death Collaborators, 2015; Roth et al., 2015). In 2013, approximately 173,000 people died from CAD worldwide, accounting for approximately 31% of all deaths. Based on the above trends, it is estimated that about 236,000 people will die from CAD by 2030 (WHO, 2011). Related reports indicate that about 50% of the occurrence and development of CAD are attributed to elevated plasma lipids (Kisfali et al., 2010; Tada, Kawashiri & Yamagishi, 2017; Waterworth et al., 2010). Numerous studies have confirmed that changes in plasma lipid levels are closely associated with the occurrence of cardiovascular risk events (Clarke et al., 2007; Stamler et al., 2000). In a large-scale epidemiological study conducted in China between 2002 and 2014, the overall prevalence of dyslipidemia in adults increased significantly. Reducing the prevalence of dyslipidemia can significantly reduce the prevalence of cardiovascular disease in a population.

Dyslipidemia is the outcome of the interaction between the environment and genetics. Previous research has demonstrated that genetic factors influence total cholesterol, triglycerides, low-density lipoprotein cholesterol, and high-density lipoprotein cholesterol (Tada et al., 2014). However, even when the cause of the rare mutation is taken into account, known genetic variants only explain 10% to 25% of dyslipidemia. This suggests that interactions between genes and the environment cannot entirely explain the pathogenesis of dyslipidemia (Natarajan et al., 2018; Willer et al., 2013). Thus, the function of epigenetic mechanisms in regulating blood lipid levels is becoming increasingly recognized.

DNA methylation is an important form of epigenetic modification. DNA methylation refers to the covalent bonding of a methyl group to the 5th carbon position of cytosine in genomic CpG dinucleotides by DNA methyltransferase (van der Harst, de Windt & Chambers, 2017). Abnormal DNA methylation refers to the specific hypermethylation or hypomethylation of gene promoters, which decreases or increases gene expression, respectively (Baylin & Ohm, 2006). DNA methylation predominantly occurs in higher animals and affects gene expression without altering the original gene base sequence. Currently, DNA methylation is the most studied epigenetic topic in the field of lipid metabolism. Epigenome-wide association studies (EWAS) have found that methylation of several genes, including *CPT1A*, *ABCG1*, and *SREBF1*, are associated with lipid metabolism. Carnitine palmitoyl transferase 1A (*CPT1A*) is a key enzyme in the B oxidation of long-chain fatty acids in mitochondria and plays an important role in blood lipid metabolism. Braun et al. (2017) found that methylation levels at two CpG sites of *CPT1A* (cg00574958 and cg17058475) were inversely correlated with triglycerides and LDL cholesterol. *ABCG1* is an important lipid homeostasis-regulating protein. Furthermore, earlier research has shown that the DNA methylation levels of cg06500161 and cg27243685 are positively correlated with triglycerides and negatively correlated with high-density lipoprotein cholesterol (Gomez-Alonso et al., 2021; Pfeiffer et al., 2015). *SREBF1* affects cholesterol metabolism by regulating the transcription of LDLR. In a previous study, increased DNA methylation at cg11024682 and cg20544516 was found to be positively correlated with dyslipidemia (Lai et al., 2016).

However, previous research has not examined the association between the methylation of other key genes of lipid metabolism and dyslipidemia. Some genes that have been linked to dyslipidemia in population-based GWAS research have not been studied. Furthermore, earlier research has primarily focused on the relationship between DNA methylation levels at individual CpG sites of genes and dyslipidemia. There are multiple CpG sites in gene promoters, however, the relationship between DNA methylation of CpG regions and dyslipidemia has not been explored. In this study, we focused on the relationship between CpG regions in gene promoter regions and lipid metabolism. The genes (*AMFR, FBXW7, INSIG1, INSIG2*, and *MBTPS1*) were found to be associated with lipid metabolism. Glutamate receptor, ionotropic, N-methyl-D-aspartate associated protein 1 (*GRINA*) was found to be associated with LDL (Chu et al., 2015). However, the molecular mechanism of *GRINA* has not been studied. The correlation between DNA methylation of these genes and lipid metabolism has not been thoroughly investigated. This study aimed to examine the relationship between the DNA methylation of these genes and the risk of dyslipidemia by comparing methylation levels between dyslipidemia and control samples.

## Materials and Methods

### Study population

Before beginning this study, we developed a research strategy based on STROBE list-case control and the Declaration of Helsinki and obtained approval from the Ethical Review Committee of the First Affiliated Hospital of Xinjiang Medical University (Additional Files 1–3). From 2012 to 2015, we enrolled 100 participants as the case group and 80 participants as the control group at the First Affiliated Hospital of Xinjiang Medical University (Additional File 1). All participants’ information was collected by experienced and trained clinicians. Before data collection, all participants signed an informed consent form. The information gathered includes age, gender, history of hypertension, history of diabetes, smoking history, *etc*.

Dyslipidemia was defined as fasting TG level ≥1.7 mmol/L, TC level ≥5.2 mmol/L, LDL level ≥3.1 mmol/L, or HDL level <1.0 mmol/L. People who had not previously received anti-inflammatory, lipid-lowering, or other anti-heart failure treatments were considered eligible for participation in the study. The exclusion criteria were as follows: 1. Those complicated with acute heart failure, malignant arrhythmia, or other heart diseases such as heart valve disease; 2. Those combined with severe cerebrovascular, liver, kidney, and lung tissue diseases; 3. Those combined with systemic infectious diseases, malignant tumors, or thyroid disease; 4. Those combined with blood, immune, endocrine, nervous system, or severe mental illness.

### DNA isolation and epigenotyping

Fasting venous blood was drawn from all participants and placed in an EDTA (ethylene diamine tetraacetic acid) anticoagulant tube. DNA was extracted from peripheral blood using commercial kits (TIANGEN Biotech, Beijing, China) and diluted with 75% ethanol before sequencing and analyzing the DNA methylation.

**Figure 1:**
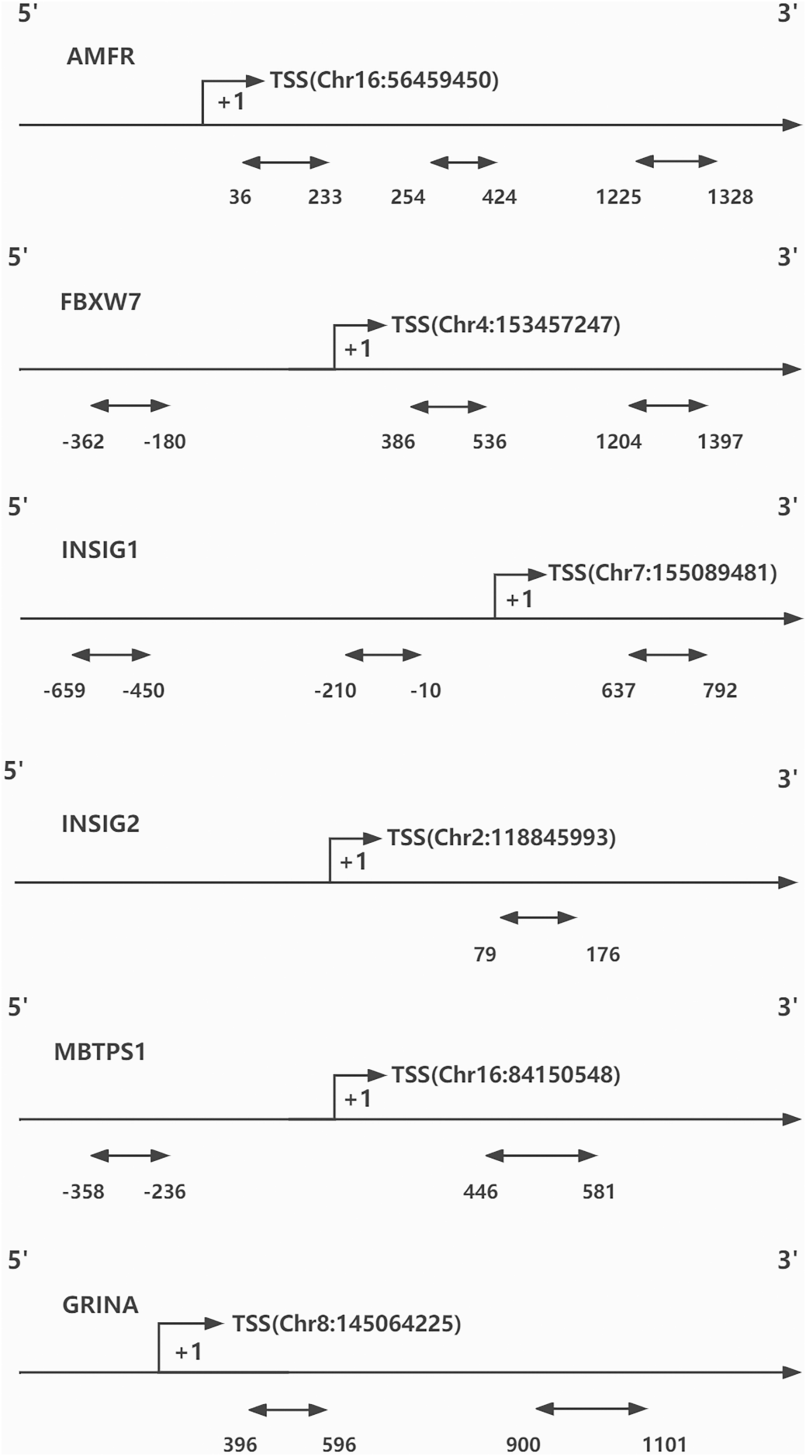
CpG sites sequenced around the promoters of genes. TSS, transcription start site.

DNA methylation was evaluated by BiSulfite Amplicon Sequencing (BSAS). The purpose of using BSAS to modify DNA is to completely convert the unmethylated cytosine in the DNA sequence into uracil, while the methylcytosine remains unchanged. The CpG islands distributed in promoters and first exon areas of *AMFR, FBXW7, INSIG1, INSIG2, MBTPS1*, and *GRINA* genes were sequenced using an Illumina MiSeq Benchtop Sequencer (San Diego, CA, USA) for methylation genotyping and analysis (Because the effect of DNA methylation on gene transcription mostly occurs in the promoter region and the first exon region). Finally, three regions from CpG islands of *AMFR*, three from *FBXW7*, three from *INSIG1*, one from *INSIG2*, two from *MBTPS1*, and two from *GRINA* were selected and sequenced (Fig. 1). Bisulfite converse the base of 1 μg genomic DNA using EZ DNA Methylation- GOLD Kit (Zymo Research, Irvine, CA, USA) before sequencing. The CpG areas examined were defined as the distance (in bp) between CpG sites and the transcription start site (TSS). The CpG site methylation level was defined as the methylated cytosine/ whole cytosine ratio. The gene methylation level was defined as the average methylation level of all detected CpG sites. The criteria for CpG site selection and the specific process of DNA methylation analysis are based on our previous research methods, which are documented in Additional File 1 (Li et al., 2020).

### Statistical analysis

All data analyses were performed using SPSS 22.0, and the Kolmogorov-Smirnov method was used to test the normality of the measurement data. Measurement data conforming to the normal distribution were expressed as mean ± standard deviation. The t-test was used to compare the means of two independent samples. The overall methylation level of each group of DNA samples was expressed as median (interquartile range). The Mann-Whitney U test was used to compare groups with non-normally distributed measurement data. Counting data were expressed as the number of cases (percentage). We applied the Chi-square test or Fisher’s exact test to examine count data. *P* < 0.05 indicates a statistically significant difference. Logistic regression analyses (OR and 95% CI) were used to determine independent risk factors or protective factors of the disease.

## Result

A total of 180 participants were included for data analysis. Table 1 shows the demographic characteristics of the study participants. The average age of the 180 participants was 59.89 ± 10.97 years old, with 60 (33.3%) of them being female. Because the degree of DNA methylation was significantly correlated with gender and age, there were no significant gender and age differences between the two groups, indicating that the research protocol was feasible. There were 100 (55.5%) participants with HTN and 44 (24.4%) with DM. The dyslipidemia group had a higher prevalence of hypertension and diabetes than the control group. The dyslipidemia group had higher levels of TC, TG, HDL, LDL, and glucose than the control group (Table 1).

**Table 1:** Comparison of basic characteristics between dyslipidemia group and control group.

Characteristics	All (*n* = 180)	Without dyslipidemia (*n* = 80)	With dyslipidemia (*n* = 100)	*P* value
Age, years	59.89 ± 10.97	58.56 ± 11.60	60.45 ± 10.38	0.148
Female, *n* (%)	60 (33.3)	28 (35)	32 (32)	0.671
HTN, *n* (%)	100 (55.5)	33 (41.25)	67 (67)	0.001
Diabetes, *n* (%)	44 (24.4)	10 (12.5)	34 (34)	0.001
TC, mmol/l	4.38 ± 1.20	3.61 ± 0.66	4.99 ± 1.19	0.001
TG, mmol/l	1.79 ± 1.05	1.11 ± 0.33	2.34 ± 1.10	0.001
HDL, mmol/l	1.20 ± 0.32	1.35 ± 0.30	1.09 ± 0.28	0.001
LDL, mmol/l	2.91 ± 1.02	2.17 ± 0.64	3.52 ± 0.85	0.001
Glucose, mmol/l	6.00 ± 2.33	5.14 ± 1.31	6.69 ± 2.72	0.001
GSP, mmol/L	2.30 ± 0.44	2.22 ± 0.38	2.36 ± 0.47	0.025
Creatinine, mmol/L	71.96 ± 20.77	71.03 ± 17.70	72.70 ± 22.98	0.593
Smoking, *n* (%)	56 (31.1)	22 (27.5)	34 (34)	0.349

**Notes:**

The Kolmogorov-Smirnov method was used to test the normality of the measurement data. The measurement data conforming to the normal distribution are expressed as the mean ± standard deviation. The comparison of the means between the two groups uses two independent samples. *t* test. The non-normally distributed measurement data used the median (interquartile range), and the Mann Whitney U test was used for comparison between groups. For counting data, it is expressed as the number of cases (percentage). Apply Chi-square test or Fishers exact test. if *P* < 0.001 will described as *P* = 0.001.

HTN, hypertension; TC, Total cholesterol; TG, Triglyceride; HDL, High-density lipoprotein; LDL, Low-density lipoprotein; GSP, Glycated serum protein.

A total of 259 CpG sites (62 in *AMFR*, 51 in *FBXW7*, 70 in *INSIG1*, 10 in *INSIG2*, 24 in *MBTPS1*, and 42 in *GRINA*) were measured in target 11 regions (the specific CPG distribution information is listed in the Additional File 4). After analyzing the CpG sites of different regions, a total of 24 CpG sites were discovered to be related to dyslipidemia. The CpG sites with differences in DNA methylation expression levels in dyslipidemia and control groups are listed. Dyslipidemia is linked to CpG sites 34 and 69 of the *GRINA* gene (Table 2).

**Table 2:** CpG site methylation of candidate genes between dyslipidemia group and control group.

Gene	Genome position	Distance 2TSS	Region position	Methylation level (median %, minimum—maximum)		*P* value
				Without dyslipidemia (*n* = 80)	With dyslipidemia (*n* = 100)	
GRINA_1	145,064,632	407	34	0.50 (0–5.29)	0.92 (0–4.82)	0.012
	145,064,667	442	69	0.85 (0–3.27)	0.60 (0–3.56)	0.023
GRINA_2	145,065,320	1,095	37	0.71 (0–25)	3.77 (0–26.92)	0.028
MBTPS1	84,150,882	−334	53	3.59 (1.36–7.18)	3.80 (2.01–6.13)	0.038
	84,150,868	−320	67	3.41 (2.11–5.64)	3.62 (155–6.71)	0.016
	84,150,784	−236	151	0.43 (0–1.16)	0.49 (0–1.05)	0.047
INSIG1_2	155,089,454	−27	41	0 (0–5)	0 (0–4.55)	0.042
	155,089,391	−90	104	1.80 (0–7.45)	1.32 (0–6.67)	0.028
	155,089,369	−112	126	1.79 (0–7.79)	2.4 (0–10.34)	0.044
INSIG1_3	155,090,156	675	66	0.57 (0–1.65)	0.67 (0–2.35)	0.024
INSIG2	118,846,072	79	28	0 (0–11.11)	0 (0–13.33)	0.039
FBXW7_1	153,456,802	445	85	5.13 (0–10.53)	4.70 (0–8.81)	0.048
	153,456,754	493	133	1.07 (0–5.26)	1.42 (0–4.50)	0.050
FBXW7_2	153,457,430	−183	30	0.43 (0–2.12)	0.52 (0–1.63)	0.025
	153,457,454	−207	54	0.75 (0–6.67)	0.97 (0.11–2.30)	0.025
	153,457,492	−245	92	0.38 (0–7.69)	0.45 (0.08–1.37)	0.028
	153,457,529	−282	129	1.77 (0–5.56)	1.87 (0.80–3.20)	0.021
	153,457,534	−287	134	1.42 (0–3.23)	1.56 (0–2.79)	0.017
AMFR_1_	56,459,028	422	39	0.49 (0–2.17)	0.73 (0–1.76)	0.003
	56,459,044	406	55	1.49 (0.27–4.18)	1.26 (0–3.27)	0.027
	56,459,196	254	207	1.55 (0.16–4)	1.86 (0.53–4.57)	0.007
AMFR_2	56,459,330	120	140	0 (0–3.85)	0 (0–4.26)	0.019
	56,459,414	36	224	0 (0–4.35)	0 (0–6.67)	0.008
AMFR_3	56,458,225	1,225	94	2.99 (0–20)	2.34 (0–7.33)	0.013

**Notes:**

The non-normally distributed measurement data used the median (interquartile range), and the Mann Whitney U test was used for comparison between groups.

Genome Position: Location of CpG site on the genome Distance. Distance 2TSS: The distance of the CpG site relative to the transcription start site on the reference genome. Region Position: The position of the CpG site on the CpG region. Methylation Level: The methylated cytosine/whole cytosine ratio was defined as the CpG site methylation level.

In our comprehensive analysis of CpG sites in distinct gene regions, we defined the average methylation level of CpG sites as the methylation level of genes. The average DNA methylation levels of *AMFR, FBXW7, INSIG1, INSIG2*, and *MBTPS1* genes were not significantly associated with dyslipidemia. DNA methylation of *GRINA* (Table 3), on the other hand, was associated with dyslipidemia. When compared with the control group, the dyslipidemia group had higher DNA methylation levels of *GRINA* (2.71 *vs* 2.44, *P* = 0.04).

**Table 3:** DNA methylation level of candidate genes between dyslipidemia group and control group.

Gene	Gene methylation level (%)		Methyl different (%)	*P* value
	Without dyslipidemia (*n* = 80)	With dyslipidemia (*n* = 100)		
AMFR	1.30 (0.36–2.07)	1.27 (0.90–2.05)	−0.03	0.77
FBXW7	1.65 (0.46–2.05)	1.63 (0.91–2.13)	−0.02	0.61
INSIG1	1.27 (0.77–1.64)	1.28 (0.75–1.76)	0.01	0.80
INSIG2	2.44 (0–11.6)	2.47 (0–6.12)	0.03	0.80
MBTPS1	2.07 (1.67–17.85)	2.1 (1.67–14.20)	0.03	0.66
GRINA	2.44 (0.424–4.43)	2.71 (0.79–5.50)	0.32	0.04

**Notes:**

The non-normally distributed measurement data used the median (interquartile range), and the Mann Whitney U test was used for comparison between groups.

Gene methylation level: The average methylation level of all detected CpG sites was defined as the gene methylation level.

The analysis of DNA methylation haplotypes of distinct genes revealed specific methylation haplotypes (Table 4). The DNA methylation haplotype with significant abundance of *GRINA* gene was ttttttttttttcttttttttttt (*P* = 0.017).

**Table 4:** The haplotypes of related genes between dyslipidemia group and control group.

Gene	Haplotype	*P* value
AMFR_1_	ttttttttttttttttctttttttt	0.003
	tcttttttttttttttttttttttt	0.005
FBXW7	ttttttttttttttttttttctt	0.019
	tttttcttttttttttttttttt	0.006
INSIG1	ttctttttttttttttt	0.010
	tttttttttttttttttttcttttttttttttttttt	0.021
	tttttttttttttttttttttttcttttttttttttt	0.046
	ttttcttttttttttt	0.013
	tctttttttttttttt	0.043
INSIG2	ttttttttct	0.012
	tttttctttt	0.012
	ttttcttttt	0.039
MBTPS2_	ttttttctttt	0.019
	tttttttttct	0.014
	tttcttttttt	0.007
	tcttttttttt	0.044
	ttctttttttt	0.019
	tcccctccctc	0.013
	tcccccccttt	0.013
GRINA	ttttttttttttcttttttttttt	0.017

**Notes:**

The non-normally distributed measurement data used the median (interquartile range), and the Mann Whitney U test was used for comparison between groups.

Haplotype: Assuming that the amplified sub sequence is “AXTXAXT”, X can be C (methylated) or T (unmethylated). If the sequencing result is “ATTCATT”, the amplified methylation haplotype is “tct”.

The methylation levels of *GRINA* are a risk factor for dyslipidemia (*P* = 0.019, OR = 1.548 95% CI [1.073–2.233]) (Table 5). Participants with higher *GRINA* methylation have a 54% increased risk of dyslipidemia. Hypertension (*P* = 0.024, OR = 2.184 95% CI [1.109–4.302]) and hyperglycemia (*P* = 0.003, OR = 1.408 95% CI [1.122–1.766]) are risk factors for dyslipidemia, which is consistent with previous research. Participants with higher blood glucose or hypertension have a 40–118% increased risk of developing dyslipidemia.

**Table 5:** Logistic regression analysis for risk factors that could affect lipid metabolism.

Characteristics	Univariate		Multivariate	
	*P* value	OR	*P* value	OR
GRINA	0.028	1.459 [1.041–2.044]	0.019	1.548 [1.073–2.233]
Sex	0.638	0.861 [0.462–1.604]		
Age	0.148	1.020 [0.993–1.048]		
Smoking	0.194	0.656 [0.347–1.240]		
Hypertension	0.001	2.807 [1.530–5.150]	0.024	2.184 [1.109–4.302]
Diabetes	0.001	3.606 [1.651–7.876]	0.241	1.701 [0.700–4.132]
Creatinine	0.591	1.004 [0.990–1.018]		
Glucose	0.001	1.549 [1.255–1.910]	0.003	1.408 [1.122–1.766]
Gsp	0.031	2.157 [1.071–4.344]	0.211	1.679 [0.745–3.782]

**Note:**

*P* < 0.05 indicates statistical difference. Logistic regression analyses (OR and 95% CI) is used to analyze independent risk factors or protective factors of the disease.

## Discussion

In recent years, various genome-wide association studies (GWAS) have discovered common mutants that affect blood lipid metabolism (Do et al., 2015; Jørgensen et al., 2014; Crosby et al., 2014). Over the last decade, researchers have discovered the specific molecular mechanisms of several plasma lipid-related genes such as *LDLR, APOB*, and *PCSK9*. Recent research has also discovered a link between the *LIMA-1* gene and lipid metabolism (Chu et al., 2015; Zhang et al., 2018). In addition to genetic inheritance, epigenetic changes are also related to lipid metabolism (Fazio & Linton, 2012). Previous research has discovered that HMG CoA Reductase (*HMGCR*) catalyzes the conversion of HMG-CoA to mevalonate, which is the rate-limiting step in cholesterol biosynthesis. Sterols promote *HMGCR* degradation *via* the ubiquitin-proteasome pathway, thus slowing down cholesterol biosynthesis. Autocrine Motility Factor Receptor (*AMFR*) is a ubiquitin ligase anchored to the ER membrane and can ubiquitinate *HMGCR. AMFR* interacts with another endoplasmic reticulum membrane protein insulin-induced gene (*INSIG*)-1 or *INSIG*-2 *via* its transmembrane domain to form a complex. Low cholesterol levels prevent *HMGCR* from binding to the *INSIG-AMFR* complex. HMGCR interacts with the *INSIG-AMFR* complex when cholesterol levels increase, resulting in ubiquitination and degradation of *HMGCR* (Song, Javitt & DeBose-Boyd, 2005; Song, Sever & DeBose-Boyd, 2005). According to previous research, membrane-bound transcription factor protease site 1 (MBTPS1) gene expression protein is a member of the *PCSK9* protease family (*PCSK1, PCSK2, FURIN, PCSK4-PCSK7, MBTPS1*, and *PCSK9*), which cleaves the target protein to activate it (Taylor, Van De Ven & Creemers, 2003). Previous research demonstrates that PCSK9 is an important protein that regulates lipid metabolism. PCSK enzymes play an important role in the progression of atherosclerosis by modulating the activity of the pre-atherosclerosis factor (Stawowy & Fleck, 2005). Glutamate receptor, ionotropic, N-methyl-D-aspartate associated protein 1 (*GRINA*) was found to be associated with LDL (Wu et al., 2018). However, the molecular mechanism of *GRINA* has not been thoroughly investigated. However previous research has indicated that only about 40–50% of changes in LDL-C levels are determined by genetic factors (Nadir & Struthers, 2011; Palacios et al., 2012). It is unknown whether the DNA methylation levels of these key lipid metabolism genes are associated with dyslipidemia.

In this study, three CpG sites in *MBTPS1*, two CpG sites in *INSIG1*, one CpG site in *INSIG2*, seven CpG sites in *FBXW7*, and six CpG sites in *AMFR* were found to be associated with dyslipidemia. In contrast to previous research, we not only examined the relationship between DNA methylation at CpG sites and dyslipidemia but also determined the relationship between overall DNA methylation at CpG regions and dyslipidemia. This study found a significant correlation between DNA methylation in the CpG region of *GRINA* and dyslipidemia. The study also demonstrated that DNA methylation of three CpG sites in the *GRINA* gene was associated with dyslipidemia. Logistic regression analysis revealed that DNA methylation of *GRINA* may increase the risk of dyslipidemia. These findings support the notion that *GRINA* DNA methylation may increase the risk of dyslipidemia. In our previous research, we discovered that DNA methylation of the *TBL2* gene is associated with hyper-low-density lipoprotein cholesterolemia. However, Hyper-LDL is only one type of dyslipidemia. The relationship between DNA methylation and dyslipidemia has not been exhaustively studied. In this study, the relationship between the DNA methylation level of the *TBL2* gene and abnormal blood lipids was examined. The DNA methylation level of the *TBL2* gene was lower in the dyslipidemia group than in the control group consistent with previous findings. At the same time, the previously discovered distinct CpG sites were also detected. Further verification of the reliability of the results is required.

DNA methylation is a pre-transcriptional modification that involves the precise addition of methyl groups to a nucleotide. DNA methylation regulates gene expression and preserves genomic integrity by interacting with modified nucleosome proteins (Jin & Liu, 2018). CpG islands around transcriptional initiation sites in the genome are allocated differently at certain stages. Typically, DNA methylation in conjunction with CG methylation inhibits gene expression (Weber et al., 2005; Song et al., 2005; Grunau, Hindermann & Rosenthal, 2000). There are two ways in which promoter methylation might inhibit gene transcription. First, physically detaching the transcription elements and gene promoter complexes. Second, histone or chromatin modifiers bind to the methyl-CpG-binding domain, thereby activating repressive machinery and causing chromatin compaction (Clouaire et al., 2010). Methylated CpG islands are biological indicators of gene suppression because the function as docking sites for methyl-binding proteins. They can primarily inhibit transcription components to gene promoters, recruit transcription inhibitors, and impede activation protein binding (Luo, Hajkova & Ecker, 2018). In our previous research, we found that the DNA methylation level of the *TBL2* gene is associated with hyper-low-density lipoprotein cholesterolemia (Li et al., 2020).

*GRINA* is a member of the *TMBIM* gene family, which codes for six proteins possessing a transmembrane BAX inhibitor motif (*TMBIM*). These genes encode calcium channels present in the Golgi, endoplasmic reticulum (ER), and mitochondria, which regulate calcium homeostasis (Lisak et al., 2015; Rojas-Rivera et al., 2012; Rice et al., 2019). According to a recent study, *GRINA/TMBIM3* modulates voltage-gated Ca V 2.2 Ca^2+^ channels in a G-protein-like manner (Mallmann et al., 2019). *TMBIMs* regulate cell death, including during ER stress by regulating calcium flow, with the majority of the proteins being anti-apoptotic (Rice et al., 2019; Liu, 2017). Recent clinical research has demonstrated that the transmembrane protein *GRINA* modulates aerobic glycolysis and promotes tumor progression in gastric cancer (Xu et al., 2018). *GRINA* was also found to be associated with elevated levels of antigliadin antibodies (AGA IgG) in subgroups of schizophrenics (Cihakova et al., 2019). Rice et al. (2019) also discovered that *GRINA* is a novel methylation quantitative trait loci associated with osteoarthritis. Previous research has associated the inhibition of calcium channels with an increase in membrane cholesterol in neuroblastoma-glioma hybrid cells. We hypothesized that *GRINA* regulates membrane cholesterol by regulating calcium channels.

In gene expression studies, the expression levels of the Nuclear Receptor Subfamily 1 Group H Member 2 gene (*NR1H2*) and GRINA expression levels are comparable across conditions. The Gene Expression Omnibus (GEO) database demonstrates the co-expression relationship between the *NR1H2* and *GRINA* genes (Bahr et al., 2013; Dobbin et al., 2005; Rieger et al., 2004; Wang, Ramrattan & Cheung, 2015). According to the reactome pathways data, *NR1H2* is associated with *VLDLR. NR1H2* is a protein-coding gene. Previous research has demonstrated that *NR1H2* is associated with biliary diseases. *NR1H2* is associated with the lipid metabolism pathway. NR1H2 activates *ABCA1* (ATP Binding Cassette Subfamily A Member 1) gene transcription by binding to the *ABCA1* gene promoter, which is mediated by PPARγ (Mogilenko et al., 2010). ABCA1 also plays a significant role in cholesterol efflux on the plasma membrane. Under conditions of high cellular cholesterol content, *NR1H2* dissociates from surface-resident *ABCA1* (cs*ABCA1*), rendering cs*ABCA1* susceptible to ubiquitination (Fu et al., 2013; Mizuno, Hayashi & Kusuhara, 2015). Previous research has shown that *ABCA1* is closely associated with lipid metabolism, and *ABCA1* in the liver is involved in the transformation of phospholipid to apolipoproteins and the formation of high-density lipoprotein cholesterol (Quazi & Molday, 2013). Overall, the up-regulation of *GRINA* gene DNA methylation influences the expression of the *NRIH2* gene, which in turn affects the expression of *ABCA1* on the plasma membrane and cholesterol efflux.

In contrast, prior research demonstrated that *NR1H2* is recruited in the promoter region of the Sterol Regulatory Element Binding Transcription Factor (*SREBF*) gene to influence the regulation of *SREBF1* (Flaveny et al., 2015). *SREBFs* are transcription factors that regulate the transcription of genes involved in lipid and cholesterol metabolism. *SREBFs* regulate cellular cholesterol homeostasis. *SREBF* cleavage activation protein (*SCAP*) and *INSIG* form a composite with *SREBF* to aid in its transport to the Golgi apparatus in sterol-depleted cells. Sterol-depleted cells have two special proteases, which can convert related enzymes into those that stimulate the amino-terminal transcription operating region of *SREBF*. The activated *SREBF* complex can then enter the nucleus and bind to the promoter of the target gene (Rawson, 2003; Walker et al., 2011). In addition, previous research has demonstrated a correlation between *GRINA* and *SREBF*. Overall, the increased DNA methylation of *GRINA* may affect the *SREBF* pathway by altering the expression of the *NR1H2* gene, and consequently, blood lipid metabolism.

*NR1H2* can induce the expression of the *IDOL* gene, and *IDOL* can reduce the level of *LDLR* by catalyzing its ubiquitination. The reduction in *LDLR* causes alterations in serum cholesterol levels (Zelcer et al., 2009). Therefore, we hypothesized that the increase in *GRINA* methylation affects the level of *NR1H2*, which, in turn, affects the level of *LDLR* and, ultimately, the change in serum cholesterol.

### Study strengths and limitations

There are several advantages to this study. One of the most significant advantages is that it is the first to discover that *GRINA* methylation is associated with dyslipidemia. This study also discovered that several *GRINA* haplotypes are associated with dyslipidemia. Furthermore, this study was the first to discover that several CpG sites in genes involved in lipid metabolism (*AMFR, FBXW7, INSIG1/2, MBTPS2*) are associated with dyslipidemia. In the future, these findings could be used to predict diseases using gene methylation. Third, this is a random case-control study, with a human subject and therapeutic application value. This study also has limitations. First, this study is a correlation study. Further functional research is required to interpret the mechanisms linking *GRINA* methylation to dyslipidemia. Second, this is a single-center study, and all participants were Han Chinese, which may limit the generalizability of the findings.

## Conclusions

The results of this study suggest that DNA methylation of *GRINA* increases the risk for dyslipidemia in humans. The DNA methylation levels of 23 CpG sites in six genes were shown to be associated with dyslipidemia, and a total of 20 DNA methylation haplotypes showed statistically significant differences between the two groups.

## Supplemental Information

10.7717/peerj.14590/supp-1 Supplemental Information 1 Checklist of case control study.

10.7717/peerj.14590/supp-2 Supplemental Information 2 Sequencing analysis.

10.7717/peerj.14590/supp-3 Supplemental Information 3 Clinical information.

10.7717/peerj.14590/supp-4 Supplemental Information 4 Sequencing raw data.

## List of abbreviations

AMFR: Autocrine motility factor receptor
FBXW7: F-Box and WD repeat domain containing 7
INSIG1: Insulin induced gene 1
INSIG2: Insulin induced gene 2
MBTPS1: Membrane bound transcription factor peptidase, site 1
GRINA: Glutamate ionotropic receptor NMDA type subunit associated protein 1
LDLR: Low Density Lipoprotein receptor
APOB: Apolipoprotein B
PCSK9: Proprotein convertase subtilisin/kexin type 9
LIMA-1: LIM domain and actin binding 1
HMGCR: 3-Hydroxy-3-Methylglutaryl-CoA Reductase
TBL2: Transducin beta like 2
TMBIM: Transmembrane BAX inhibitor motif containing
NR1H2: Nuclear receptor subfamily 1 group H member 2
ABCA1: ATP binding cassette subfamily A member 1
SREBF: Sterol regulatory element binding transcription factor
SCAP: SREBF cleavage activation protein
IDOL: Myosin regulatory light chain interacting protein
CAD: Coronary Artery Disease
GWAS: Genome-wide association studies
EDTA: Ethylene diamine tetraacetic acid
BSAS: BiSulfite amplicon sequencing
TSS: Transcription start site
TC: Total cholesterol
TG: Triglyceride
HDL: High-density lipoprotein
LDL: Low-density lipoprotein
GSP: Glycated serum protein

## Additional Information and Declarations

### DNA Deposition

The following information was supplied regarding the deposition of DNA sequences: The sequencing data is available at SRA: PRJNA819883. http://www.ncbi.nlm.nih.gov/bioproject/819883.

### Data Availability

The following information was supplied regarding data availability: The raw data is available in the Supplemental Files.
